# Synthetic opioid contamination in Brazil’s illicit drug market: an emerging and underdetected risk

**DOI:** 10.1016/j.lana.2026.101587

**Published:** 2026-08-01

**Authors:** Mariana Campello de Oliveira, João Maurício Castaldelli-Maia

**Affiliations:** a Instituto Perdizes (Iper), do Hospital das Clínicas da Faculdade de Medicina da Universidade de São Paulo, São Paulo, SP, 05021-001, Brazil; b Faculdade de Medicina da Universidade de São Paulo (FMUSP), Departamento de Psiquiatria, São Paulo, 01246-903, Brazil

In June 2026, Brazil’s National Secretariat for Drug Policy and Asset Management (SENAD) issued a nationwide alert following the laboratory confirmation of fentanyl in cocaine samples seized in the state of Espírito Santo, with compatible findings also reported in São Paulo.^1^ Although the number of documented cases remains limited, the significance of this finding extends beyond its frequency. For the first time, Brazilian authorities have officially confirmed the presence of a highly potent synthetic opioid adulterating one of the most widely consumed and distributed illicit substances in the country.

Brazil’s position within global drug markets gives this issue particular relevance. The country occupies a central role in international cocaine trafficking routes and is one of the world’s largest cocaine consumers markets.^2^ At the same time, it has historically remained peripheral to the heroin trafficking routes that contributed to the development of large illicit opioid markets elsewhere.^2^ This context may have shielded Brazil from earlier waves of the opioid crisis observed in North America.^3^ Opioid consumption in Brazil has traditionally been driven predominantly by prescription opioids,^4^ and preliminary data from the Third Brazilian National Alcohol and Drug Survey (LENAD III, 2025), publicly released by the Brazilian Ministry of Justice and Public Security, indicate that opioid use has increased more than nine-fold over the past decade.^5^ However, recent findings suggest that synthetic opioids are not necessarily restricted to contexts traditionally associated with opioid use.

Forensic systems and early warning mechanisms have documented the circulation of new psychoactive substances in the Brazilian drug market. Among them are nitazenes, a class of synthetic opioids whose potency can equal or exceed that of fentanyl.^6^ A report published in 2025 by the United Nations Office on Drugs and Crime (UNODC) in partnership with Brazil’s Ministry of Justice documented their presence in multiple Brazilian states.^7^ Data from São Paulo indicated that most recent seizures involving synthetic opioids contained nitazenes, often identified in association with synthetic cannabinoids.^8^ These observations suggest that synthetic opioids are no longer confined to a single substance or a single context of drug use.

Perhaps the most concerning aspect of these findings is that individuals may be exposed to synthetic opioids without intending to consume them. Both fentanyl identified in cocaine and nitazenes detected in products containing synthetic cannabinoids can expose individuals to opioids without their knowledge. In a setting where opioid use remains relatively uncommon compared with other illicit drugs,^4^ many individuals have little or no opioid tolerance and do not expect to be exposed to substances capable of causing severe respiratory depression.^9^ Beyond increasing the risk of overdose, this pattern may complicate clinical recognition of opioid toxicity, as patients may present to emergency services reporting only the substance they believed they had consumed.

The epidemiological extent of this phenomenon remains uncertain. Toxicological surveillance in Brazil faces important limitations, and more than three quarters of overdose death records lack specification of the substance involved.^3^ As fentanyl and nitazenes are not routinely investigated in many clinical and forensic settings, opioid-related poisonings and deaths may remain unidentified or be attributed solely to the primary drug consumed. The true burden of synthetic opioid exposure may therefore be substantially underestimated.

Current reports do not support the conclusion that Brazil is facing an opioid crisis comparable to those observed in the United States, but they do point to the emergence of a risk that warrants close monitoring.^10^ More than isolated findings, the recent detection of fentanyl in cocaine and nitazenes in synthetic cannabinoids calls for strengthened surveillance of illicit synthetic opioids and a better understanding of their presence in drug markets not traditionally associated with this class of substances. While the window for proactive action remains open, experience from countries further along this trajectory suggests it may not stay open for long.

## Contributors

MCO: Conceptualisation, investigation, writing—original draft, writing—review & editing. JMCM: Conceptualisation, writing—review & editing. Both authors approved the final version of the manuscript.

## Declaration of interests

We declare no competing interests.
